# Studies on the Impact of the Photoinitiator Amount Used during the PVP-Based Hydrogels’ Synthesis on Their Physicochemical Properties

**DOI:** 10.3390/ma15176089

**Published:** 2022-09-02

**Authors:** Magdalena Kędzierska, Magdalena Bańkosz, Piotr Potemski

**Affiliations:** 1 Department of Chemotherapy, Medical University of Lodz, Copernicus Memorial Hospital of Lodz, 90-549 Lodz, Poland; 2 Department of Materials Engineering, Faculty of Materials Engineering and Physics, Cracow University of Technology, 37 Jana Pawła II Av., 31-864 Krakow, Poland

**Keywords:** hydrogels, *Aloe vera* juice, vitamin C, sorption capacity, wettability, tensile strength, percentage elongation, simulated physiological liquids, surface morphology, photopolymerization

## Abstract

In recent times, a great interest is directed to developing biomaterials incorporated with various therapeutical substances which may enhance them with new properties and thus increase their application potential. In this work, polyvinylpyrrolidone (PVP)-based hydrogels modified with *Aloe vera* juice and vitamin C and differing in the amount of the photoinitiator used during their synthesis were developed. Analysis of hydrogels included characterization of their chemical structure via FT-IR spectroscopy, sorption properties, wettability, surface morphology, behavior in simulated physiological liquids and mechanical properties. Finally, hydrogels’ cytotoxicity towards L929 murine fibroblasts using MTT reduction assay was additionally verified. It was demonstrated that as the amount of the photoinitiator used during the synthesis of hydrogels increased, the smoother their surface and the higher their hydrophilicity. Next, the greater the amount of the photoinitiator, the lower is the percentage elongation of the hydrogel and the greater the hardness. In turn, the swelling ability of hydrogels depended strongly on the type of the absorbed liquid—swelling ratios of samples in distilled water were 24% higher than in SBF, 18% higher than in Ringer liquid, and 32% higher than in hemoglobin wherein the amount of the photoinitiator did not affect this property. Additionally, hydrogels were stable and did not degrade in simulated physiological liquids. The only changes in pH of the incubation media were probably caused by the active substances release from hydrogels which was also confirmed via a lesser intensity of the absorption band on FT-IR spectra corresponding to the functional group occurring in compounds included in *Aloe vera* juice. Importantly, the viability of fibroblasts incubated with developed materials was at least 86%. Thus the hydrogels, due to their properties, seem to show application potential to be used for biomedical purposes, e.g., as innovative dressing materials.

## 1. Introduction

The role of dressing materials is mainly to absorb the wound exudate, to constitute a barrier preventing the penetration of microorganisms from the external environment into the wound as well as provide adequate wound hydration [1,2]. For this purpose, such solutions are applied as, e.g., sterile gauze pads or topical antimicrobial ointments and creams. Nonetheless, they are often not effective enough and thereby the wound healing process is long-lasting and persistent for the patient [3]. Thus, one of the main challenges of the scientists is to develop multifunctional dressings accelerating wound healing processes [4]. Among currently developed dressing materials, various characteristics may be mentioned, such as self-healing [5], self-removal [6], stimuli-responsive [7,8] or dressings with monitoring capacity [9,10].

Particular attention is directed to the development of dressings modified with various modifiers enhancing such materials with new properties. For example, a modification of the dressings with an antibacterial agent leads to the preparation of the materials preventing or dealing with bacterial infections [11,12]. Such materials have been reported, e.g., by Wang et al. Here, membranes incorporated with copper boron–imidazolate framework conducive to healing of wounds with bacterial infections have been developed [13]. Another solution is the introduction of a modifying agent promoting wound healing process into the dressing material. Such additives may be, for example, Aloe vera juice showing both antimicrobial properties and accelerating healing processes [14] or vitamin C which also promotes wound healing [15].

In this paper, synthesis and characterization of PVP-based hydrogel polymers containing *Aloe vera* juice and vitamin C have been presented. The materials have been prepared via UV radiation. To our knowledge, hydrogels with such a composition and prepared via a photopolymerization process have not yet been described. Importantly, the impact of 2-hydroxy-2-methylpropiophenone—as photoinitiator—on the properties of PVP-based hydrogels modified with active substances also has not yet been discussed. A photoinitiator is used to initiate the photopolymerization process. Thus, its amount and concentration may affect the properties of final polymers. For example, Kamoun et al. discussed the impact of the irradiation time and the photoinitiator (photoinitiating system consisting of carboxylated camphorquinone acting as a photoinitiator, diphenyliodonium tetrafluroborate acting as an accelerator and folic acid used as amine coinitiator) concentration on hydrogels’ crosslinking performance. It was demonstrated that as the photoinitiator concentration and the irradiation time increased, the hydrogel surface porosity decreased and hydrogels’ storage modulus increased [16]. In turn, Geever et al. proved that as the photoinitiator concentration increased, the tensile strength of hydrogels decreased and their swelling ability increased [17]. Next, Holmes et al. reported that the concentration of the photoinitiator used for preparation of poly(ethylene glycol) and collagen-based hydrogels affected their swelling properties, storage modulus and the cytotoxicity towards selected cell lines [18]. The impact of the type of the photoinitiator used during the hydrogel synthesis was additionally confirmed by Yang et al. [19].

The studies described in this paper aimed at a detailed analysis of the physicochemical properties of the hydrogels wherein the main attention was focused on defining the impact of the amount of the photoinitiator used during the synthesis on the characteristics of final materials. The analyses performed included incubation studies determining the behavior of the hydrogels in simulated physiological liquids (simulated body fluid SBF, Ringer liquid, hemoglobin and distilled water as a reference liquid) and characterization of the chemical structure of hydrogels via Fourier transform infrared (FT-IR) spectroscopy. Importantly, the materials were tested spectroscopically both before and after the incubation to verify the impact of such environments on samples’ structures. Moreover, sorption capacity of hydrogels in simulated physiological liquids as well as their surface morphology using Scanning Electron Microscopy (SEM) was also determined. Next, the wettability of prepared materials and their mechanical properties including tensile strength, percentage elongation and hardness were also characterized. Finally, hydrogels’ cytotoxicity towards L929 cell lines using MTT reduction assay was also verified.

## 2. Materials and Methods

### 2.1. Materials

Polyvinylpyrrolidone (PVP, average molecular weight 58,000, powder), 2-hydroxy-2-methylpropiophenone (photoinitiator, d = 1.077 g/mL, 97%), diacrylate poly(ethylene glycol) (crosslinking agent, average molecular weight M_n_ = 700 g/mol, PEGDA) and L-Ascorbic acid (vitamin C, ACS reagent, ≥99%) were bought in Sigma Aldrich (Saint Louis, MO, USA). *Aloe vera* juice was purchased from Herbal Pharmaceuticals (Kraków, Poland).

### 2.2. Synthesis of Hydrogel Materials

Hydrogels were obtained by means of the photopolymerization process. The polymer matrices were based on polyvinylpirrolidone wherein vitamin C and Aloe vera juice were applied as modifiers. 2-hydroxy-2-methylpropiophenone was used as a photoinitiator while diacrylate poly(ethylene glycol) (PEGDA) acted as a crosslinking agent. The reaction mixtures consisted of adequate amounts of PVP solution, vitamin C solution, modifiers, crosslinking agent and photoinitiator. They were poured onto the Petri dishes and treated with UV radiation for 120 s. As a source of radiation, Emita VP-60 lamp (λ = 320 nm, power: 180 W, manufacturer: Famed, Lodz, Poland) was applied. The exact compositions of the prepared materials are presented in Table 1.

The materials after synthesis were dried at 37 °C for 24 h and subsequently subjected to investigations aimed at determining their physicochemical properties. Importantly, particular attention was paid to verifying the impact of various amount of photoinitiators used during the synthesis of hydrogels on their characteristics.

### 2.3. Assessment of the Sorption Capacity of Hydrogels

One of the most characteristic features of hydrogels is the ability to reversibly absorb various liquids. Therefore, developed materials were analyzed in terms of this property while attention was focused on discussing the impact of various factors on their sorption capacity. Factors considered included hydrogels’ chemical compositions (various amounts of photoinitiator), the type of the absorbed liquid (distilled water, simulated body fluid—SBF—isotonic to human blood plasma, Ringer liquid—infusion liquid and 2% hemoglobin solution) as well as the swelling period (1 h, 24 h, and 48 h). Importantly, the study was performed at room temperature. Firstly, dry hydrogel samples weighing 1.0 g were immersed in 50 mL of all tested media and remained in this environment for 1 h. After this time, samples were separated from the solutions, an excess water (free water) was removed using a paper towel and hydrogels were weighed again their swollen state. Then, samples were placed again in tested media and the procedure was repeated after 24 h and 48 h. In order to determine hydrogels’ swelling ability, the swelling ratios (α) were calculated using the following Equation (1):

(1) $$\alpha =\frac{\left(m-m_{0}\right)}{m_{0}}$$

where: *α*—swelling ratio, g/g; *m*—mass of swollen hydrogel, g/g; and *m*_0_—mass of dry hydrogel, g.

### 2.4. Evaluation of the Impact of Hydrogels on Simulated Physiological Liquids (Incubation Studies)

Considering the potential application of hydrogels for biomedical purposes it is important to verify their behavior and/or tendency to degradation in environments simulating physiological body fluids. For the study, the same liquids as in the case of sorption measurements were employed. Hydrogel samples weighing approximately 1.0 g were placed in 50 mL of tested liquids for 8 days while every two days the temperature and the pH of the incubation media were measured. A multifunctional CX-701 pH-meter (Elmetron, Zabrze, Poland) was used for measurements. The study was conducted at room temperature.

### 2.5. Characterization of the Chemical Stucture Hydrogels via FT-IR Spectroscopy

The next analysis included verifying the presence of characteristic functional groups in the structure of hydrogels using FT-IR spectroscopy. This study was used also to verify whether the incubation of hydrogels in simulated physiological liquids affected their structure; therefore, the samples were subjected to the analysis before and after the incubation. The study was performed using a Thermo Scientific Nicolet iS5 FT-IR spectrophotometer (Thermo Fisher Scientific, Waltham, MA, USA). FT-IR spectra of hydrogel samples were recorded within the wavenumber range 4000–500 cm^−1^ (32 scans and the resolution 4.0 cm^−1^).

### 2.6. Analysis of Hydrogels’ Surface Morphology Using SEM Technique

An important aspect of the research was also to characterize the surface morphology of the hydrogels and to verify whether the amount of the photoinitiator used during their synthesis affected this feature. For this purpose, the Jeol 5510LV scanning electron microscope (Jeol Ltd., Tokyo, Japan) was applied. Firstly, dry hydrogel samples were sputtered with gold and then subjected to the microscopic analysis which was carried out at ambient temperature.

### 2.7. Wettability of Hydrogels

Performed investigations included also determining whether obtained hydrogels show hydrophilicity or hydrophobicity. For this purpose, their wettability was characterized wherein two liquids were used during the study, i.e., water (polar solvent, ultra-high quality) and diiodomethane (non-polar solvent). The hydrophilicity/hydrophobicity of the analyzed sample is reflected via the value of the contact angle. In order to determine the value of the contact angle (defined also as the wetting angle) between the surface of the selected liquid and the outline of the hydrogel surface, the sessile drop method was used. The Drop Shape Analysis system (DSA 10Mk2, Kruss, Germany) was applied for this purpose.

The method applied allowed determination of the surface free energies (polar, dispersive and total free energy) for each hydrogel sample. For the calculation of the particular components of the surface free energy the Owens–Wendt method was applied. This method is based on the assumption that the interactions occurring between the molecules of two substances within their surface layer are equal to the geometric average of interactions within each substance. The polar and dispersive components of analyzed substances may be calculated using the following Equations (2) and (3):

(2) $${\left(\gamma_{S}^{D}\right)}^{0.5}=\frac{\gamma_{d}\left(cos\theta_{d\;}+1\right)-\sqrt{\frac{\gamma_{d}^{P}}{\gamma_{w}^{P}}\gamma_{w}\left(cos\theta_{w}+1\right)}\;}{2\;⎣\sqrt{\gamma_{d}^{D}-\sqrt{\gamma_{d}^{P}\frac{\gamma_{w}^{D}}{\gamma_{w}^{P}}}}⎦}$$

(3) $${\left(\gamma_{S}^{P}\right)}^{0.5}=\frac{\gamma_{w}\left(cos\theta_{w}+1\right)-2\;\sqrt{\gamma_{S}^{D}\gamma_{w}^{D}}\;}{2\;\sqrt{\gamma_{w}^{P}}}$$

where *γ_S_^D^* and *γ_S_^P^* are dispersive (*D*) and polar (*P*) components of surface free energy of tested samples, *γ_d_* is surface free energy of non-polar liquid (diiodomethane, *d*), *γ_d_^P^* and *γ_d_^D^* are polar and dispersive components of non-polar liquid surface free energy, *γ_w_* is surface free energy of polar liquid (water, *w*), *γ_w_^P^* and *γ_w_^D^* are polar and dispersive components of polar liquid surface free energy, *θ_d_* and *θ_w_* are contact angles of diiodomethane (*d*) and water (*w*).

### 2.8. Studies on Mechanical Characteristics of Hydrogels

In the course of the research, mechanical properties of the hydrogels were also characterized. The analyses were conducted in accordance with the ISO 527-2 type 5A [20] and ISO 37 type 2 [21] standards. Firstly, the paddle-shaped samples were prepared using the ZCP020 manual blanking press. Such samples were subsequently fixed between the jaws of the universal testing machine (Shimadzu, Kyoto, Japan). During the analysis, the jaws of the apparatus moved apart from each other resulting in the sample elongation. The study was carried out until the hydrogel sample was broken. Such experiments allowed measurement of the stress-strain dependence while two parameters for each sample were determined, i.e., the percentage elongation (*A*) and the tensile strength (*R_m_*). Parameter A was calculated by means of Equation (4) and parameter B using Equation (5); both equations are presented below:

(4) $$A=\frac{100\times \left(I_{u}-I_{0}\right)}{I_{0}}$$

(5) $$R_{m}=\frac{F_{m}}{S_{0}}$$

where: $F_{m}$—the maximum strength, $S_{0}$—the cross-sectional area of analyzed material before the measurement, $I_{u}$—the measuring length after the hydrogel was broken, $I_{0}$—the measuring length of the hydrogel sample before the measurement.

Apart from the percentage elongation and the tensile strength of hydrogels, their hardness was also determined. For this purpose, the Insize (Insize Inc., Loganville, GA, USA) Shore A Hardness tester was applied. The hydrogel sample was pressed against the base by means of the hardness tester which proceeded simultaneously with hammering a needle-like indenter in the tested material. After establishing the balance between the material and the pressure, the appropriate hardness value was read from the tester. 10 measurements were performed for each sample. The analysis was conducted at room temperature.

### 2.9. Assessment of the Cytotoxicity of Hydrogels via MTT Reduction Assay

Considering the application of the developed materials for biomedical purposes it is necessary to verify their impact on the viability of selected cell lines. The basic study which allows for such a verification includes determining the cytotoxicity using MTT reduction assay. This assay provides information on potential toxicity of the tested materials, thus indicating whether they can be directed to further, more advanced study. MTT assay is an enzymatic assay verifying the metabolic activity of tested cells. It involves defining the ability of a selected enzyme (mitochondrial dehydrogenase) secreted by properly functioning cells to convert soluble MTT reagent (3-(4,5-dimethylthiazol-2-yl)-2,5-diphenyltetrazolium bromide; tetrazolium salt) into insoluble crystals of dark-blue formazan. Formed crystals may be next dissolved in an organic solvent (e.g., dimethyl sulfoxide (DMSO)) wherein the solution obtained may be next analyzed spectrophotometrically. The concentration of formazan crystals solution corresponds to the amount of the mitochondrial dehydrogenase present in the tested environment and thus to the amount of properly functioning cells. The study was performed using L929 murine fibroblasts. The culture of these cells as well as the whole procedure of the MTT assay were described in more detail in our previous article [22].

## 3. Results and Discussion

### 3.1. Results of the Measurements of the Sorption Capacity of Hydrogels

**Figure 1:**
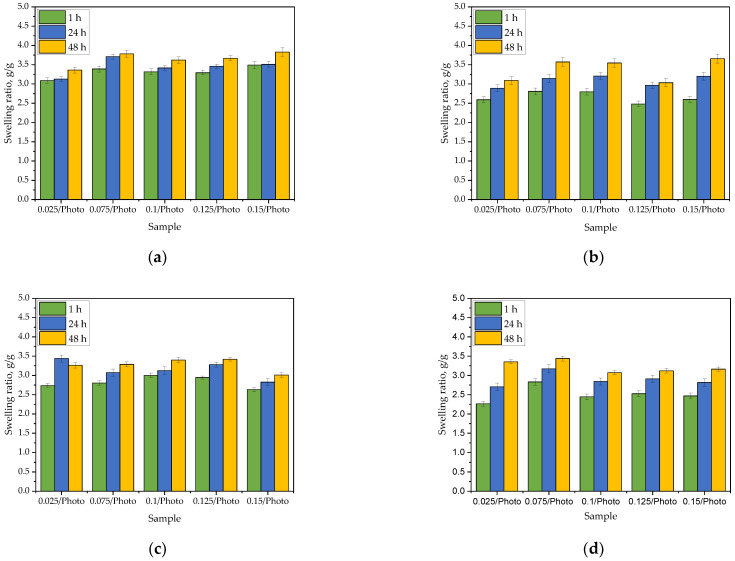
Results of the analysis of hydrogels’ sorption capacity in distilled water (**a**), SBF (**b**), Ringer liquid (**c**), and hemoglobin (**d**).

In order to determine whether the amount of the photoinitiator used during the photopolymerization affected the sorption properties of hydrogels, the swelling ratio (α) of these materials was determined. Bar charts presenting values of α calculated for each sample tested in each swelling medium during specific time period are presented below in Figure 1. The study was performed in three replicates for each sample, the results are presented as mean values ± the standard deviations (represented by the error bars).

The sorption ability is one of the basic properties of hydrogel materials. Hydrogel takes a form of a three-dimensional polymer network capable of absorbing large quantities of various liquids. Such a process is possible due to the presence of functional groups within the hydrogel polymer chains which are able to dissociate. As a result of the repulsion of ions with the same charge forming during the dissociation (for example, adjacent COO^−^ ions forming as a result of the dissociation of carboxylic groups), an increase in the distance between the polymer chains of a polymer matrix is observed. As a consequence, an increase in the volume of free spaces between these chains also takes place. These free spaces may be filled with the absorbed liquid which is, in turn, reflected in a greater sorption capacity of the hydrogel.

All obtained materials exhibited sorption capacity, and no significant impact of the photoinitiator amount on the hydrogel swelling was observed. For example, after 1 h of swelling in distilled water, the values of the swelling ratio [g/g] for particular samples were as follows: sample 0.025/Photo −3.0876 ± 0.0552; sample 0.075/Photo −3.388 ± 0.0636; sample 0.100/Photo −3.314 ± 0.0604; sample 0.125/Photo −3.292 ± 0.0533 and sample 0.150/Photo −3.492 ± 0.0785, respectively. Thus, any dependence between the amount of the initiator used during the photopolymerization process and the swelling ratio of obtained hydrogel was not reported. The same was observed for other tested media. There are no other works in which the impact of the amount of 2-hydroxy-2-methylpropiophenone on swelling properties of PVP-based hydrogels has been discussed. Studies on the impact of the photoinitiator concentration on hydrogels’ swelling ability were performed, e.g., by Geever et al. In this paper it was demonstrated that as the concentration of the photoinitiator (4-(2-hydroxyethoxy)phenyl-(2-hydroxy-2-propyl)ketone (Irgacure 2959) increased, the swelling ability of hydrogels clearly increased. For example, the percentage swelling after 2 h of the sample obtained using 0.01% wt. photoinitiator was approximately 15% while for the sample prepared using 1% wt. photoinitiator the swelling was 20%. In turn, after 48 h of swelling, the percentage swelling for the same samples were approximately 23% and 27%, respectively [17]. In presented studies, such differences have not been reported which is probably due to the too small differences in the amounts of photoinitiator between individual hydrogel samples.

On the other hand, the type of the swelling medium strongly affected the swelling of the analyzed materials. The highest swelling ratios were reported for distilled water (after 1 h of the study, the values of these parameters calculated for the same samples in distilled water were approx. 24% higher than their values in SBF, 18% higher compared to the swelling ratios calculated for Ringer liquid, and 32% higher than in hemoglobin). Distilled water penetrates into the polymer matrix to a greatest extent. This liquid does not contain any ions which might increase the crosslinking degree of the hydrogel reducing at the same time the volume of free spaces available for absorbed liquid. As a result, the swelling ratios calculated for samples tested in distilled water were the highest. In turn, in the case of the other tested media containing ions, their penetration into the hydrogel matrix was lower. The lowest values of α were determined for hemoglobin which is related to the complex structure of this compound. Moreover, it was also observed that the values of the swelling ratios increased with time wherein the biggest increase in the mass of all swelling materials was observed during the first hour of the study. This is consistent with the results presented in other works [23,24].

### 3.2. Incubation of Hydrogels in Simulated Physiological Liquids

**Figure 2:**
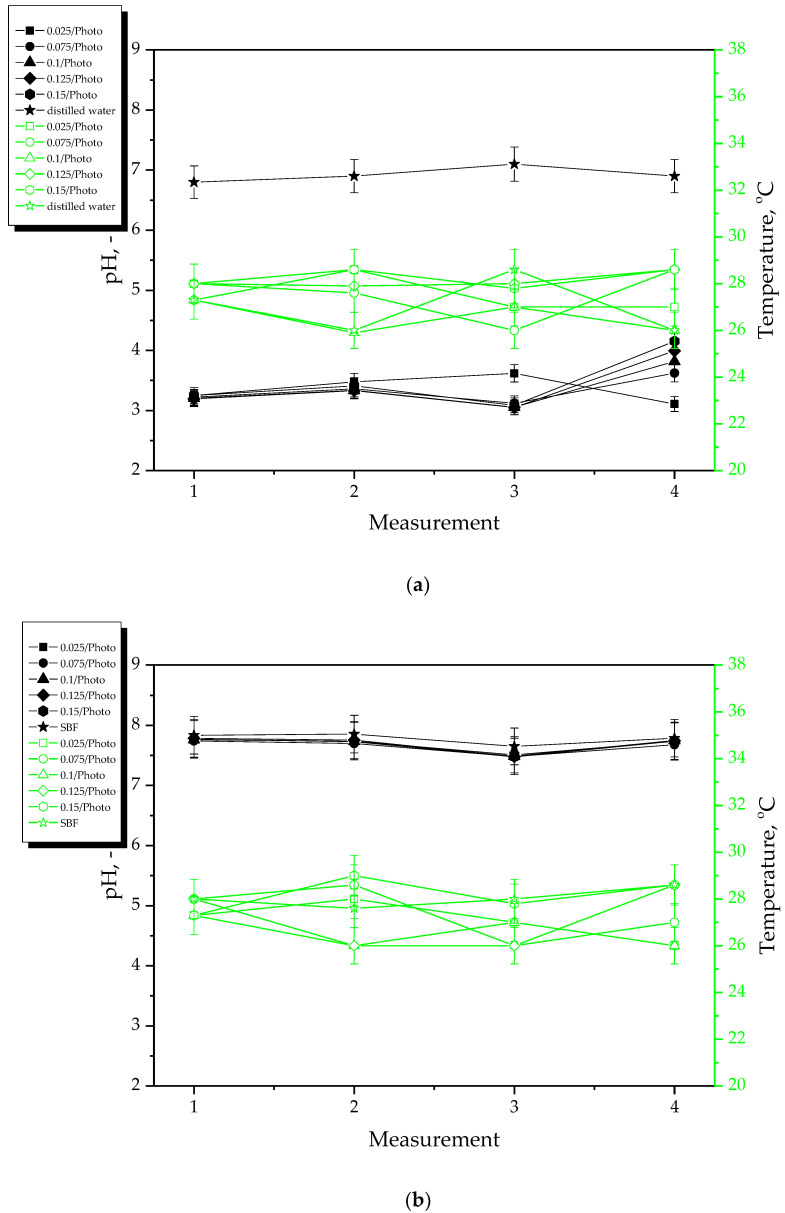
Results of incubation of hydrogels in distilled water (**a**) and SBF (**b**).

**Figure 3:**
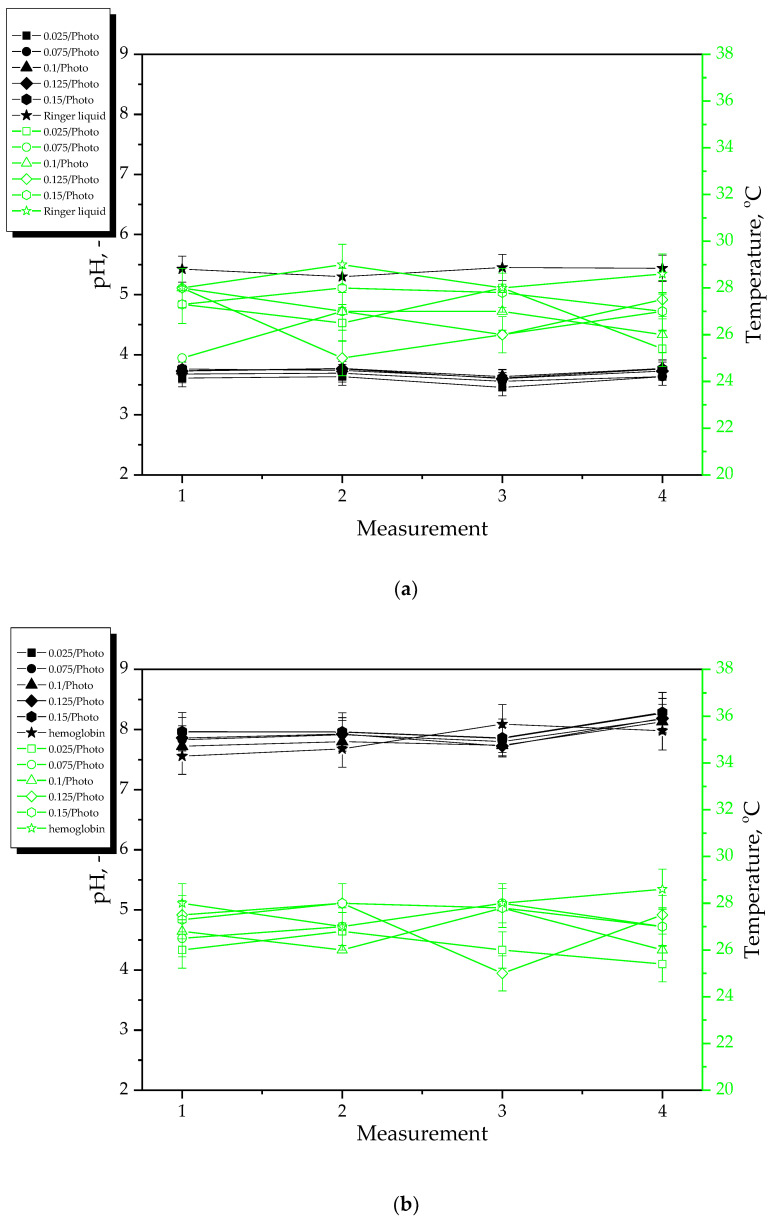
Results of incubation of hydrogels in Ringer liquid (**a**) and hemoglobin (**b**).

Results of incubation studies performed in distilled water, SBF, Ringer liquid and hemoglobin are presented below in Figure 2 and Figure 3. The study was performed in three replicates for each sample; the results are presented as mean values ± the standard deviations (represented by the error bars).

During 8-day incubation of hydrogel samples, the pH and the temperature of the incubation liquids were measured at two-day intervals. Based on the results of the studies, a clear pH change of Ringer liquid and distilled water was observed. Such a sharp pH decrease at the beginning of the study may indicate the release of the active substances (i.e., *Aloe vera* juice and vitamin C) from the hydrogel matrix. This, in turn, may cause the acidification of the incubation media. Such a phenomenon did not occur in the case of samples incubated in SBF and hemoglobin which is related to the composition of these liquids. These results are consistent with previously discussed results of swelling studies where it was demonstrated that the sorption of hydrogels in exactly these liquids was the lowest. Moreover, limited penetration of liquids related to their compositions may also reduce the release of the active substances from hydrogels in these liquids. For example, in the case of SBF containing Ca^2+^ ions, the formation of additional crosslinks between the polymer chains of hydrogel network takes place which may limit the penetration of liquid into the incubated hydrogel. This, in turn, is reflected in reducing the release of *Aloe vera* juice and vitamin C from the hydrogel which, on the other hand, affects the stability of this material during its incubation. A similar phenomenon as in the case of SBF may also be observed in the case of hemoglobin with a heme-containing protein structure wherein the polypeptide chains of amino acids included in this protein may constitute an additional limitation. Thus, it may be clearly concluded that the type of the incubation medium affects strongly the processes occurring within the incubated hydrogel samples. Importantly, the amount of the photoinitiator used during the synthesis of these polymers does not affect significantly the interactions between the material and the incubation medium. Any significant differences between the results of measurements during the incubation of hydrogels obtained using different amounts of this reagent have not been reported.

### 3.3. Verification of the Impact of Hydrogels’ Incubation in Simulated Physiological Liquids on their Structure via FT-IR Spectroscopy

**Figure 4:**
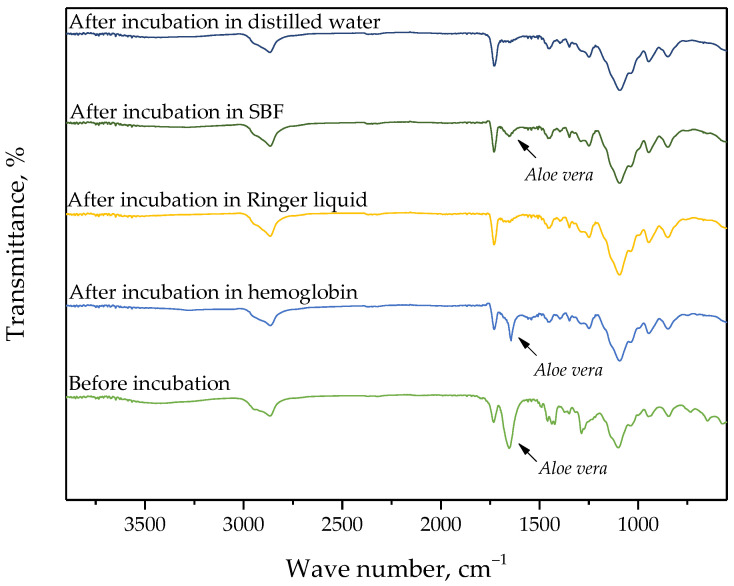
FT-IR spectrum of sample 0.025/Photo.

**Figure 5:**
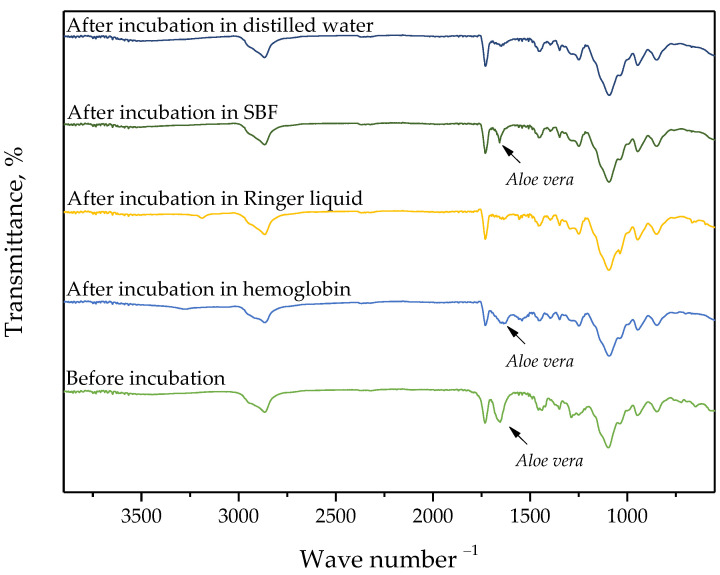
FT-IR spectrum of sample 0.100/Photo.

**Figure 6:**
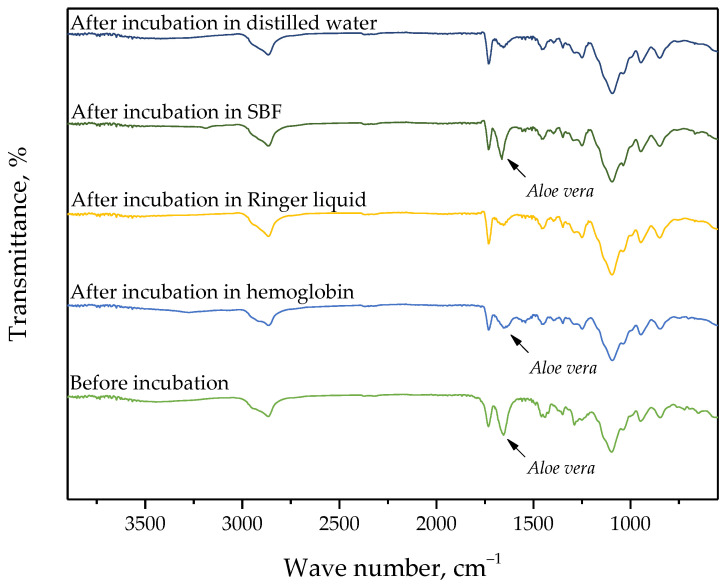
FT-IR spectrum of sample 0.150/Photo.

In the case of selected hydrogels, the spectroscopic analysis was also performed. For the study, samples were prepared using the lowest (0.025/Photo) and the highest (0.150/Photo) amount of the photoinitiator as well as the “middle” sample, i.e., obtained using 0.100 mL of this reagent (sample 0.100/Photo). The FT-IR spectra of the analyzed samples are shown in Figure 4, Figure 5 and Figure 6.

For all analyzed materials, no significant changes—e.g., a disappearance of the absorption bands on the FT-IR spectra—which could indicate the degradation of hydrogel sample in the incubation medium have been observed. On the obtained FT-IR spectra, the absorption bands characteristic for functional groups occurring in the structure of PVP, i.e., the main component of the polymer matrix, were identified. For all samples, the occurrence of the absorption bands characteristic for -CH_2_ group and C-H bond was observed at 2895 cm^−1^ and 1450 cm^−1^, respectively [25]. Furthermore, the band at 1276 cm^−1^ typical for C-N bond occurring in the structure of PVP was also seen [26]. Next, the absorption bands corresponding to the functional groups occurring in the structure of the crosslinking agent were identified, i.e., the band at 1103 cm^−1^ deriving from the stretching vibrations of C-O group and at 1730 cm^−1^ characteristic for C=O group [27,28]. FT-IR spectra of all samples were similar to each other. Importantly, a clear change was observed for the absorption band at 1665 cm^−1^. This band is characteristic of vibrations of C=O groups occurring both in the structure of polymer components and in the structure of compounds included in the *Aloe vera* juice [29]. The decrease in the intensity of this band was reported in the case of hydrogel samples incubated in distilled water and Ringer liquid. This is probably due to the release of this additive in the mentioned incubation media, which was additionally confirmed by the change in pH of these liquids noticed during the incubation studies. Thus, the results of the analysis indicated that the developed materials show the ability to release the active substance in selected incubation liquids.

### 3.4. Characteization of Hydrogels via SEM Technique

**Figure 7:**
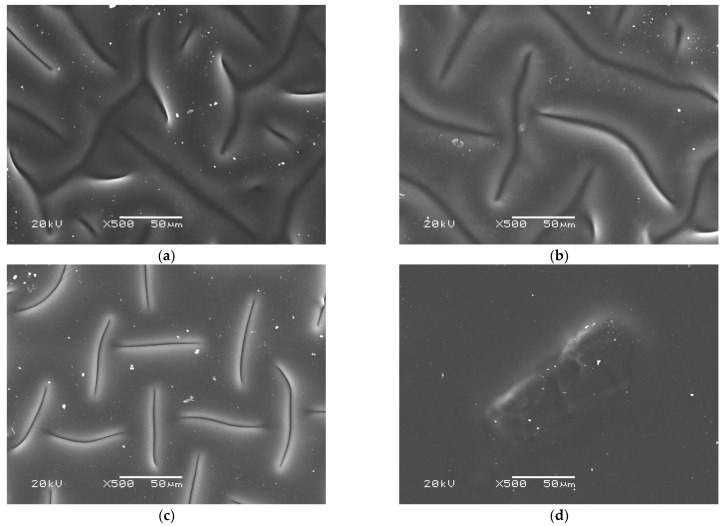

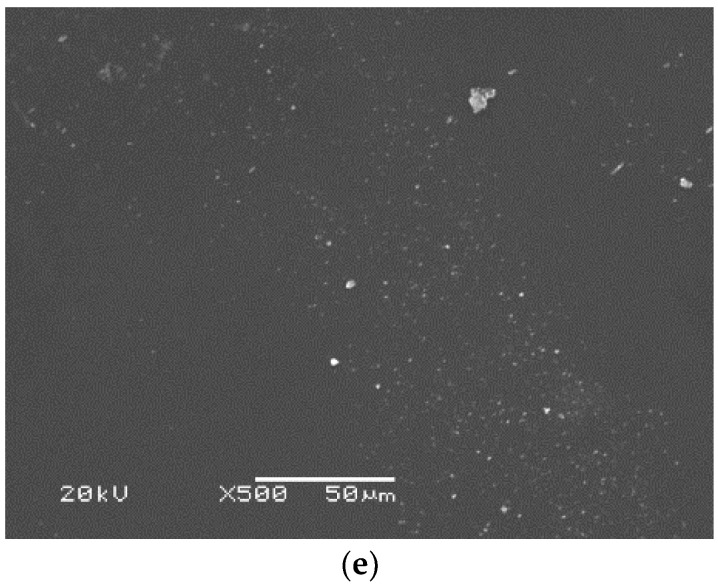
SEM image of sample 0.025/Photo (**a**), 0.075/Photo (**b**), 0.100/Photo (**c**), 0.125/Photo (**d**) and 0.150/Photo (**e**).

In order to characterize the surface morphology of developed hydrogels, SEM technique was employed. Obtained images are presented below in Figure 7.

Based on the presented SEM images, it may be concluded that the amount of the photoinitiator used during the synthesis of hydrogels strongly affected their surface morphology. The photopolymerization process is initiated via the decomposition of the photoinitiator into free radicals. The use of a large amount of this reagent results in the simultaneous initiating of this process in many sites of the reaction mixture. As a consequence, a compact polymer structure with a high crosslinking density is formed. The resulting hydrogel matrix is characterized by a significantly smoother surface compared to the matrix obtained using lower amount of the photoinitiator. In this case, the structure of the polymer network consists of a smaller number of long chains, and on the surface of such a material, numerous depressions may be observed. The greater the amount of the photoinitiator used during the photopolymerization, the smoother sample’s surface may be observed. This is clearly visible in Figure 7. The most heterogeneous surface was determined for sample 0.025/Photo while the smoothest one was reported for sample 0.150/Photo.

### 3.5. Results of Studies on Hydrogels’ Wettability

**Figure 8:**
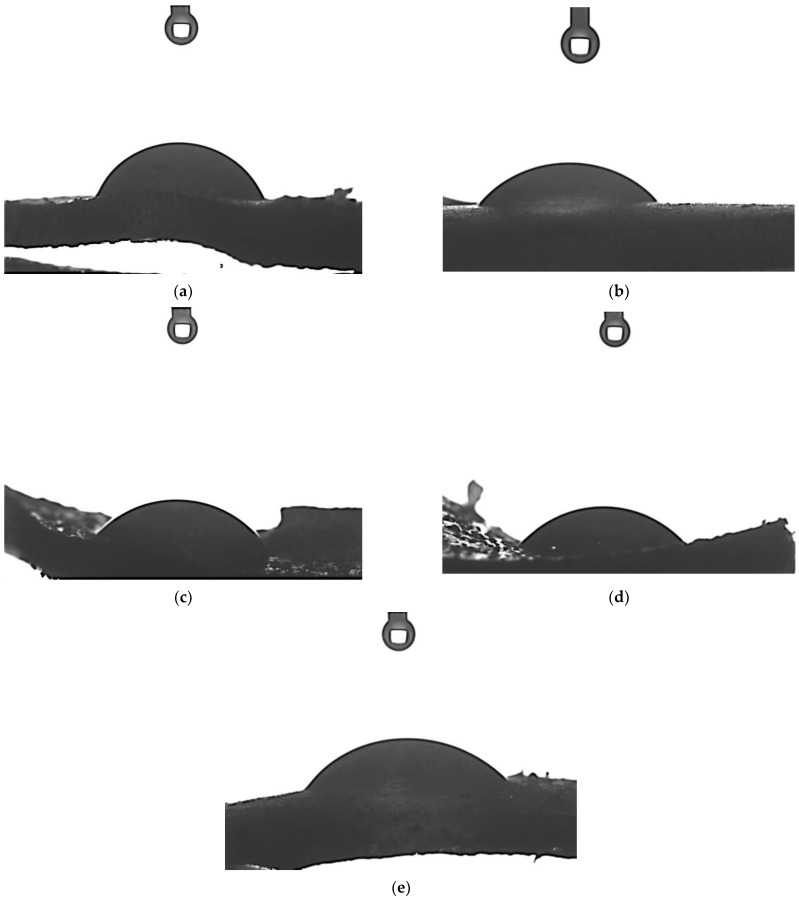
Images showing the wettability of sample 0.025/Photo (**a**), 0.075/Photo (**b**), 0.100/Photo (**c**), 0.125/Photo (**d**) and 0.150/Photo (**e**).

The next analysis concerned the verification of the hydrophilicity of developed hydrogels. For this purpose, their contact angles were determined. Results of these investigations are presented in Table 2 and in Figure 8.

Cell adhesion and proliferation on a selected surface is strictly related to its adequate preparation and specific properties. The surface wettability of a given biomaterial is correlated with the adhesion of cellular proteins which, in turn, has a significant impact on the behavior of cells. Cells usually adhere to surfaces with contact angles within the range 40–70°. The relatively hydrophilic surfaces promote cell differentiation and proliferation while the superhydrophilic surfaces (contact angle less than 5°) negatively affect cell growth [30]. Likewise, superhydrophobic surfaces may interfere with the proliferation process. For example, Cantini et al. reported that a superhydrophobic surface results in a change in the conformation of fibronectin, thus worsening the cell surface adhesion [31]. In turn, a positive impact of hydrophilic surfaces on cell growth was demonstrated by Torres et al. They proved that the advanced proliferation of cells adhering to a given biomaterial takes place on the hydrophilic surfaces [32].

In the case of all analyzed hydrogel samples, the values of contact angles were below 90° which indicates the hydrophilic nature of their surfaces. Thus, it may be concluded that the materials showing such properties may constitute a promising surface for cell growth. This, in turn, is a meaningful advantage of developed hydrogels considering their potential application for biomedical purposes. The decrease in the contact angle, i.e., an increase in a sample’s hydrophilicity, was observed with an increase in the amount of the photoinitiator used during the synthesis of hydrogels. As it was mentioned previously, the photopolymerization process is initiated by the decomposition of the photoinitiator. In the case of the use of a large amount of this compound, a hydrogel matrix with a smooth surface is observed which has been confirmed in a previous subsection concerning the results of SEM imaging. On the smoothed surface of such a material a drop of water spread much more, as evidenced by the lower contact angles compared to their values determined for samples obtained with lower amount of photoinitiator (e.g., hydrogel preparing with the lowest amount of photoinitiator—sample 0.025/Photo—is characterized by a surface with a contact angle of 24.8° greater than sample 0.150/Photo). Moreover, the photoinitiator applied (i.e., 2-hydroxy-2-methylpropiophenone) has a hydroxyl group in its structure which may interact with water via the formation of hydrogen bonds, and as a consequence improve the adhesion of a water drop to the material surface. Thus, it was concluded that an increase in the amount of the photoinitiator during the synthesis of hydrogels enhances the hydrophilicity of the surface of these materials.

### 3.6. Mechanical Properties of Hydrogels

**Figure 9:**
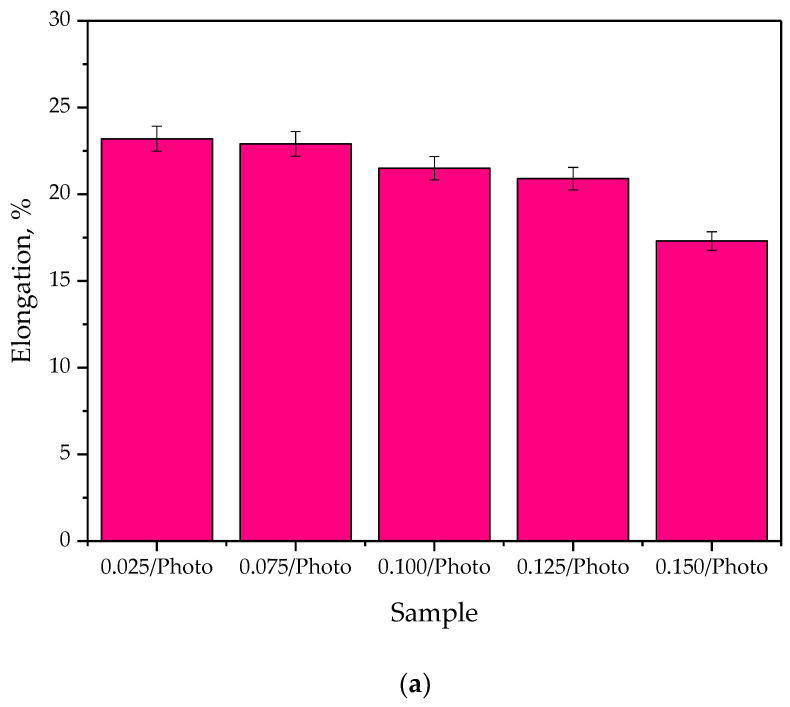

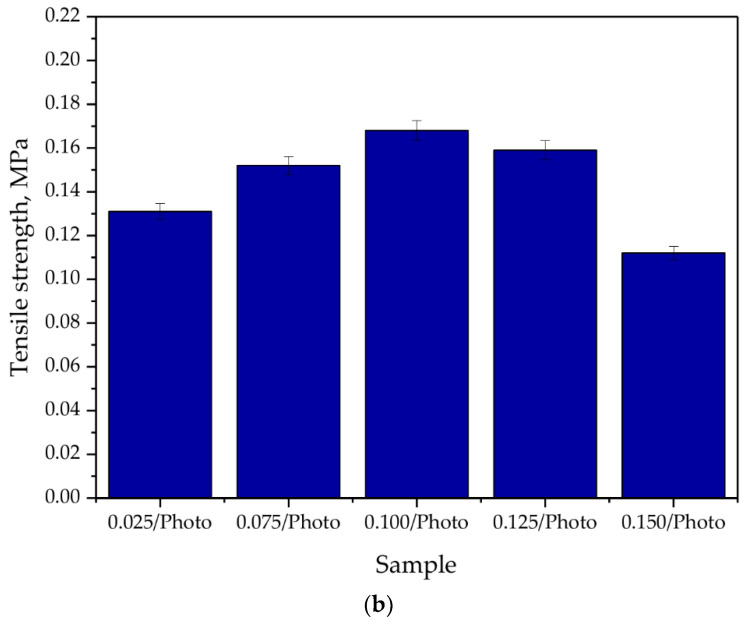
Results of mechanical studies of hydrogels showing their percentage elongation (**a**) and the tensile strength (**b**).

**Figure 10:**
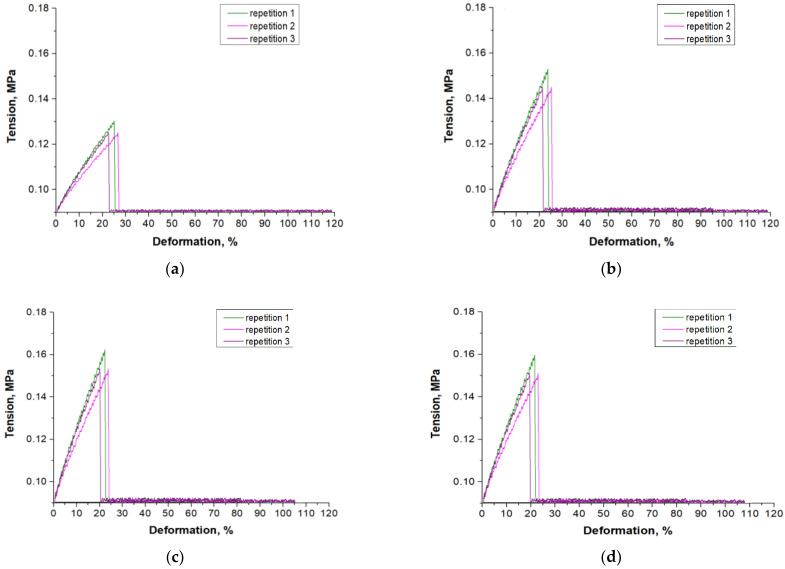

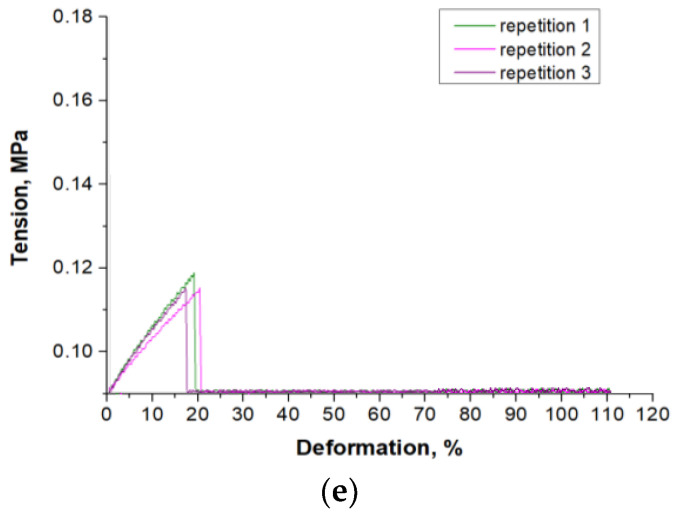
The stress-strain curves of hydrogel samples: 0.025/Photo (**a**), 0.075/Photo (**b**), 0.100/Photo (**c**), 0.125/Photo (**d**) and 0.150/Photo (**e**).

Results of the mechanical studies presenting the tensile strength and the percentage elongation of each tested hydrogel sample are shown in Figure 9 while in Figure 10 the stress-strain curves of all hydrogel samples are presented. The study was performed in 3 replicates for each sample, and the results of the analysis are shown as average values of all measurements ± standard deviations (SD, presented as error bars).

Based on performed experiments it may be concluded that the amount of the photoinitiator introduced into the reaction mixture during the photopolymerization process affected the mechanical properties of the hydrogels. As the amount of this reagent increased, the percentage elongation of hydrogels decreased. As was mentioned previously, the use of a large amount of photoinitiator results in simultaneous initiation of photopolymerization in many sites of the reaction mixture. This, in turn, leads to the formation of many short polymer chains. Such a polymer network shows high crosslinking density due to which such material has a compact structure. Therefore with an increasing amount of photoinitiator used for the synthesis of hydrogels, there is a decrease in the percentage elongation of these polymers under the tension applied and simultaneous increase in their tensile strength. However, too large an amount of this reagent results in obtaining an over-compact and consequently fragile structure. The sample obtained using 0.100 mL of photoinitiator showed the highest tensile strength while adding more of it worsened this mechanical property of the obtained material. As a result, the tensile strength of sample 0.100/Photo was 0.168 MPa while in the case of samples 0.125/Photo and 0.150/Photo, the values were 0.159 MPa and 0.112 MPa, respectively.

There are no other works in which the impact of the amount of 2-hydroxy-2-methylpropiophenone on such mechanical properties as the tensile strength and the percentage elongation of PVP-based hydrogels has been discussed. There are some investigations focusing on the impact of the concentration of the other photoinitiators on hydrogels’ mechanical properties. For example, Wiley et al., concluded that as the photoinitiator concentration increased, the elasticity of hydrogels also increased [33]. Nonetheless, the tested hydrogels contained other reagents and the synthesis conditions were also different than the ones presented here; thus, it is difficult to compare the results of these studies.

**Figure 11:**
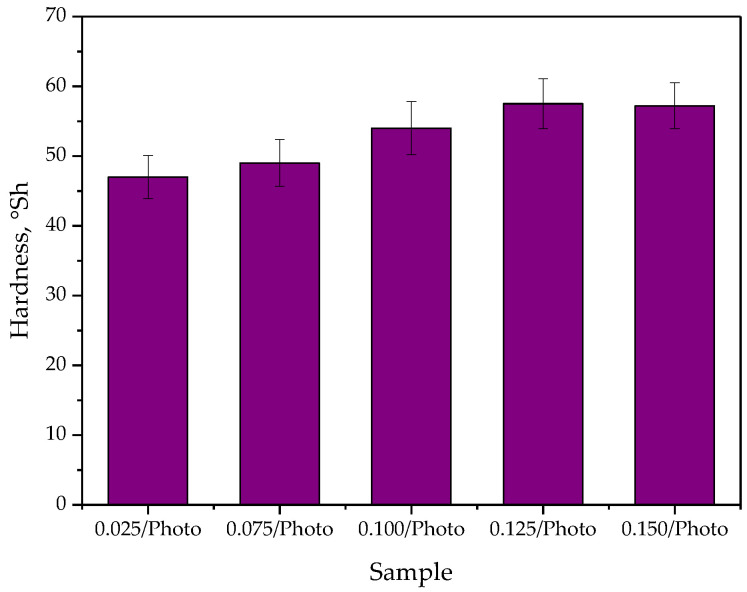
Results of hydrogels’ hardness analysis.

Next, in Figure 11, the results of studies on hydrogels’ hardness are presented. The study was performed in 10 replicates for each sample, and the results of the analysis are shown as average values of all measurements ± standard deviations (SD, presented as error bars).

The performed study included measurement of the hardness of the developed hydrogels. This property is meaningful in terms of considering the hydrogels for application as dressing materials. Hardness of such dressing affects its adhesion to the skin and thus to the surface of the wound. In the case of insufficient hardness, the dressing may stick to the affected area, and impede the healing process. On the other hand, the removal of such a dressing may disturb the continuity of the newly formed tissue [34,35].

As may be observed in Figure 11, the developed materials showed hardness within the range 47.0 ± 3.1–57.5 ± 3.6. Thus, based on the Shore Type A hardness scale it may be concluded that obtained hydrogels belong to the group of medium-soft materials. Importantly, it may also be reported that the greater the amount of the photoinitiator in the hydrogel, the greater its hardness. This is due to the previously described increase in the compactness of the hydrogel structure. The mentioned compactness is caused by the presence of a large amount of photoinitiator in the reaction mixture, and thus the initiation of photopolymerization in several places which, in turn, results in obtaining a material consisting of many short polymer chains.

### 3.7. Analysis of Hydrogels’ Cytotoxicity Using MTT Reduction Assay

**Figure 12:**
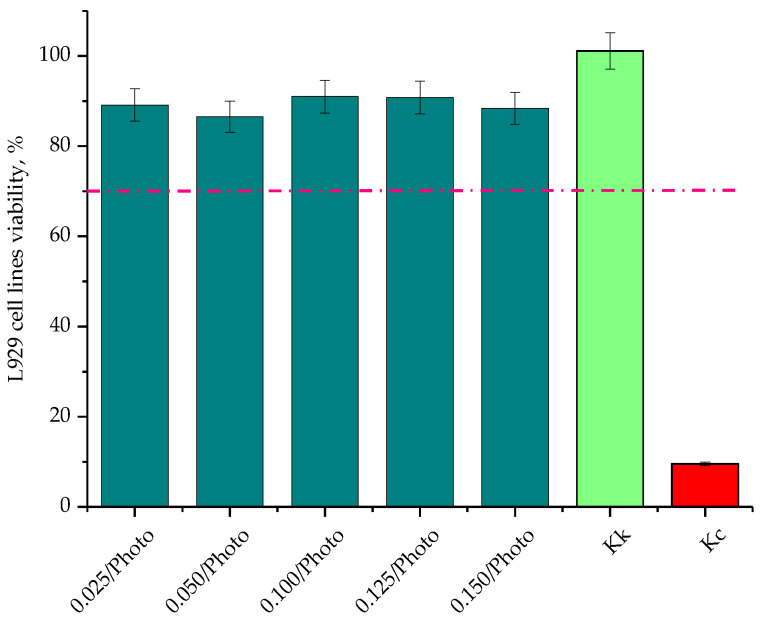
Cytotoxicity of the hydrogels towards L929 murine fibroblasts (the pink dotted line indicates 70% cell viability below which the tested material is considered as cytotoxic).

The cytotoxicity analysis was performed in triplicate for each hydrogel sample. Moreover, the study was also conducted for reference samples, i.e., Kk (positive control, cells incubated without the tested hydrogels) and Kc (negative control, cells incubated with 1% phenol solution, i.e., a well-known cytotoxic reagent). Results of the investigations are presented in Figure 12 as average values from the measurements with error bars representing the standard deviations (SD).

The cytotoxicity analysis was performed in line with an appropriate standard concerning the biological evaluation of medical devices [36]. The material is defined as cytotoxic when the viability of the cells incubated for 24 h with this material is below 70%. According to this statement, it may be clearly concluded that all the tested hydrogel samples showed no cytotoxicity towards L929 murine fibroblasts. The viability of these cells during incubation with the hydrogels was within the range of 86.54% ± 3.46–91.01% ± 3.64. Therefore, it may be reported that developed materials may be directed to further, more advanced biological investigations.

## 4. Conclusions

No dependence was observed between the amount of the photoinitiator used during the synthesis of hydrogels and their swelling ability. These properties were different depending on the absorbed liquid—the highest swelling ratios were calculated for distilled water, with values 24% higher than in SBF, 18% higher than in Ringer liquid, and 32% higher than in hemoglobin

Hydrogel samples were stable in simulated physiological liquids. The only changes in pH of incubation media resulted probably from the release of the active substances—i.e., *Aloe vera* juice and vitamin C—from hydrogel matrices.

On the FT-IR spectrum of hydrogels after incubation in simulated physiological liquids, there was less intensity of the absorption band characteristic for C=O group occurring in the structure of compounds included in Aloe vera juice which may indicate the release of this additive from the hydrogel matrix during its incubation. This confirms conclusions drawn based on the incubation studies.

The amount of the photoinitiator used during the hydrogels’ synthesis affected their surface morphology. The more there was of this reagent, the smoother the hydrogel surface.

The greater the amount of the photoinitiator, the lower was the contact angle (so greater hydrophilicity) of UV-photopolymerizable hydrogels, the lower was the percentage elongation and the greater was the hardness.

All hydrogels showed no cytotoxic properties towards L929 murine fibroblasts. The viability of these cells during the 24 h-incubation with tested materials was within the range 86.54% ± 3.46–91.01% ± 3.64.

## Figures and Tables

**Table 1:** Compositions of hydrogels.

Sample	15% PVP Solution, mL	*Aloe vera* Juice, wt.% *	5% Vitamin C Solution, wt.% *	Photoinitiator, mL	Crosslinking Agent, wt.% *
0.025/Photo	7	45	30	0.025	32
0.075/Photo				0.075	
0.100/Photo				0.100	
0.125/Photo				0.125	
0.150/Photo				0.150	

* amounts given in relation to the main component of hydrogel matrices, i.e., PVP.

**Table 2:** Parameters determined during the wettability analysis.

Sample	Contact Angle		Surface Free Energy		
	Water, °	Diiodomethane, °	Polar, mJ/m^2^	Dispersive, mJ/m^2^	Total Free Energy, mJ/m^2^
0.025/Photo	78.55	28.83	2.63	47.20	49.82
0.075/Photo	76.50	32.13	3.68	44.72	48.40
0.100/Photo	74.45	22.45	3.59	48.88	52.47
0.125/Photo	68.50	37.95	8.37	38.84	47.21
0.150/Photo	53.75	36.64	17.69	35.71	53.40

## Data Availability

Data sharing is not applicable for this article.
